# iORandLigandDB: A Website for Three-Dimensional Structure Prediction of Insect Odorant Receptors and Docking with Odorants

**DOI:** 10.3390/insects14060560

**Published:** 2023-06-15

**Authors:** Shuo Jin, Kun Qian, Lin He, Zan Zhang

**Affiliations:** College of Plant Protection, Southwest University, Chongqing 400716, China; js0510@email.swu.edu.cn (S.J.);

**Keywords:** insect odorant receptors, insect-specific odorants, ligand docking, database

## Abstract

**Simple Summary:**

Smell is an important sense for insects. The olfactory sense of insects is involved in their feeding, mating, egg-laying, predation avoidance, and communication behaviors. The use of insect-specific odorants to control insect behavior is an important control method. Here, we built iORandLigandDB, a platform for the batch prediction of insect-specific odorants based on artificial intelligence technology. The 3D structure of existing ORs in insects and the docking data with relevant odorants can be retrieved from the database.

**Abstract:**

The use of insect-specific odorants to control the behavior of insects has always been a hot spot in research on “green” control strategies of insects. However, it is generally time-consuming and laborious to explore insect-specific odorants with traditional reverse chemical ecology methods. Here, an insect odorant receptor (OR) and ligand database website (iORandLigandDB) was developed for the specific exploration of insect-specific odorants by using deep learning algorithms. The website provides a range of specific odorants before molecular biology experiments as well as the properties of ORs in closely related insects. At present, the existing three-dimensional structures of ORs in insects and the docking data with related odorants can be retrieved from the database and further analyzed.

## 1. Introduction

The olfactory sense of insects is involved in their feeding, mating, egg-laying, predation avoidance, and communication behaviors [1,2,3]. Interference with the olfactory sense can prevent insects from locating host plants, mating, and laying eggs, thereby reducing their population density to achieve effective insect control, which is the current focus of insect chemical ecology research [4,5]. Many olfactory proteins are involved in the process of olfactory recognition. Among these proteins, odorant receptors (ORs) have been the most extensively studied. Each OR complex is believed to be a heterotetramer composed of Orco subunits and odorant tuning receptor subunits ORx. These subunits are 7-transmembrane (7-TM) proteins that form heteromeric odor-gated ion channels composed of subunits of a ligand-specific (“tuning”) receptor and a co-receptor, Orco [6,7,8]. Odorant receptor (OR) proteins can recognize odorants transmitted by odorant-binding proteins in lymph fluid, convert related chemical signals into neuroelectric signals, and then transmit them to the insect nerve center, thereby affecting the behavior of insects [9]. These proteins play a key role in the identification of odorants in the insect olfactory mechanism. One important research topic in reverse chemical ecology is to screen active substances affecting insect behavior by using proteins as the targets and odorants as ligands so as to develop attractants or repellants [10]. However, the screening for odorants is generally laborious and time-consuming, and can hardly obtain ideal results. Moreover, the progress of the experimental structure determination of insect odorant receptor family proteins is very slow. To date, there is only one experimentally resolved structure in the OR family proteins, the Orco protein from *Apocrypta bakeri* [11]. Therefore, it is urgent that we develop some methods for the large-scale and effective screening of insect odorants [12].

In recent years, the rapid development of technologies for sequencing, particularly proteome and genome sequencing, has contributed to the generation of large amounts of protein sequence data [13]. The advancement of technologies for the large-scale and rapid identification of protein functions has been driven by the challenge of efficiently analyzing protein functions amidst the accumulation of massive data. With the further development of deep-learning-related algorithms, more algorithms that only use protein molecular sequence data to estimate the three-dimensional structure of proteins have been developed, which have a higher accuracy and can be used to realize the large-scale and accurate functional analysis of proteins [14,15,16,17].

Here, we constructed a website capable of predicting the three-dimensional structure of insect ORs using Alphafold2, and used the predicted three-dimensional structure for molecular docking with common volatile odorants in plants. The website allows the query of sequences, secondary structure data, related references, three-dimensional structure, and docking information of insect ORs. The database collects the information of 4426 ORs of all insects currently available in the NCBI, 46 ligands and their information in PubChem, and 119,367 docking interactions between ORs and ligands. The users can browse, download, and access the information of insect ORs and the ligand data from the database. In addition, users can directly obtain the visualized secondary structure, three-dimensional structure, and docking posture of the ligands and ORs directly from the webpage, as well as perform a series of operations and labeling to record the screenshot of the structure of interest. The database also allows the users to browse structural data from an atomic perspective, such as atomic distance and bond angle, to gain a comprehensive understanding of the relevant information. More importantly, the database allows the users to submit their own sequences and use the database’s computing resources for the free prediction of three-dimensional protein structure and protein–ligand docking. The database can also widely receive sequence information related to insect ORs, providing a channel for users to add more OR sequences, and will be constantly expanded in the future.

## 2. Materials and Methods

### 2.1. Data Sources

A total of 4473 annotated amino acid sequences of insect ORs with unknown protein structures were collected from National Center for Biotechnology Information (NCBI) database. Specifically, first, we searched for articles that experimentally determined insect odorant receptor sequences. Then, we obtained the measured insect odorant receptor sequences from the articles. Finally, we searched and downloaded the corresponding sequence files from the NCBI database. After structure prediction, the data of three-dimensional structure and secondary structure of these amino acid sequences were obtained. In addition, 46 validated pheromone small molecules were collected based on previous reports (Table 1) [18,19,20,21,22,23,24], and their structures and information were downloaded from the PubChem database. The above data were integrated into a ligand database, which was used as a virtual screening ligand library for the three-dimensional structural model of proteins to obtain the protein–ligand docking data (iORandLigandDB). In addition, we will continue to expand the data in the future, as well as enhance the accuracy of our predictive model and promptly update the database accordingly.

### 2.2. Data Processing

A total of 151 sequences with amino acid deletion were corrected in the amino acid sequence library. Homology alignment was performed using NCBI Blast+ v2.12.0 [25,26,27,28,29,30,31,32,33,34]. The makeblastdb command was used to build the alignment database with the remaining 4322 sequences and the blastp command was employed to align the error sequence from the database. When the return result had an identity value greater than 90%, homologous substitution for amino acid deletion in the sequence was carried out; when the value was lower than 90% or the sequence was the same after replacement, the sequence was deleted. Finally, 47 sequences were deleted, and a total of 104 corrected sequences were obtained. As a whole, 4426 sequences were retained at last (see Tables S1 and S2 for details).

### 2.3. Prediction of Secondary Structures and Three-Dimensional Structures

Alphafold2 [15] was used to predict the three-dimensional structure of ORs. The 4426 obtained amino acid sequences were used as input, and default parameters were taken to predict the three-dimensional structure of 15 amino acid sequences as one group by the named pipeline. The reliability of the model was judged by the output global pLDDT value, and the model with the highest global pLDDT value was selected from the five models as the three-dimensional structural model for use in the database.

The following is an explanation for using pLDDT to evaluate the reliability of the model:

1. The model of pLDDT > 90 is considered highly reliable. Due to its high reliability, it should be suitable for any application. It is very helpful for the analysis of protein structure and function.
2. The model of pLDDT between 70 and 90 is considered to have high reliability in backbone prediction.
3. The model of pLDDT < 70 is considered to have very low reliability or even considered to be unreliable. It should be applied with caution. The lower the pLDDT value, the lower the reliability.

In addition, RCSB MAXIT v11.100 (https://sw-tools.rcsb.org/apps/MAXIT, accessed on 21 May 2022) with default parameters was also used to convert the PDB file to CIF file for component input, and PDBe Molstar v1.2.0 [35] components were integrated to visualize the three-dimensional structure of ORs in the database.

Based on the predicted three-dimensional structure, the secondary structure of ORs was predicted and visualized. The DSSP algorithm of dssp v3.0.0 [36,37] was used to calculate and identify the co-ordinate data sets of protein three-dimensional structure, so as to obtain the secondary structure of each amino acid sequence. Then, the native binary version of ESPript3 [38] was used to obtain the secondary structure files that can be displayed in the database and visualized. Default parameters were used for dssp and ESPript3.

### 2.4. Virtual Screening of Ligands

The integrated 46 ligands were used as a ligand library to perform virtual screening of OR ligands. The mk_prepare_ligand.py in Meeko v0.3.3 [39,40] was employed to convert SDF-format ligand files to PDBQT format for ligand docking. In addition, visualization of secondary structures and three-dimensional structures was also performed. The secondary structure map was downloaded from PubChem database and integrated with NGL Viewer v1.0.0 [41,42] to display the three-dimensional structure of ligand molecules, such as atomic distance and bond angle.

The prepare_receptor command in ADFR Suite v1.0 [43,44] was used for dehydration, hydrogenation, and charge adjustment of the receptor. Then, fpocket v4.0 [45] was employed to predict the binding pocket of ORs, and command line operation was used to prepare the docking configuration file. Finally, AutoDock Vina v1.2.3 [46] was used for ligand docking with ORs to obtain the docking data. The affinity (kcal/mol) was taken as the reliability evaluation index, and the model with the lowest affinity value was selected from the prediction results as the docking display model in the database. The docking posture was also visualized.

### 2.5. Prediction of Binding Regions and Transmembrane Domains

We used DeePTMHMM v1.0.18 [47] to predict the transmembrane domains of 4426 amino acid sequences. The predicted transmembrane domains were aligned with the predicted binding pockets to search for the binding regions. Finally, we determined whether the ligand-binding major region of each sequence was in the transmembrane domain, inside the membrane, or outside the membrane.

### 2.6. Verification of Docking Posture

We verified the docking posture using visual molecular dynamics (VMD) [48]. Firstly, we downloaded the experimentally verified structure and corresponding ligand data from PDB. We randomly selected 50 out of 678 filtered insect odorant receptor data as the validation data. Secondly, we employed our own method to predict the docking posture of the three-dimensional structure and the ligand. Finally, we used VMD to align the experimental structure with the predicted structure to find the root-mean-square deviation (RMSD) value. We examined the value of RMSD to determine whether our structure is reliable.

### 2.7. Structure Prediction and Ligand Virtual Screening Services

Structure prediction and ligand screening have been automated in the database. The ASGI in Django Channels v3.0.4 (https://github.com/django/channels/, accessed on 21 July 2022) was used to establish the communication between web front-end and back-end server, and realize the creation of sequence files in the back-end server when the front-end sends the sequence information. Then, Celery v5.2.1 (https://github.com/celery/celery, accessed on 22 July 2022) was used to receive sequence file and create asynchronous task. Redis-Server v6.0.9 (https://redis.io/, accessed on 25 July 2022) was used as the resulting back end and the Alphafold2 structure computation and ligand docking process were integrated. Finally, the resultant file and evaluation parameters were passed back to the front-end by WebSocket. Free computing resources are provided for users.

### 2.8. Database Implementation

**Figure 1:**
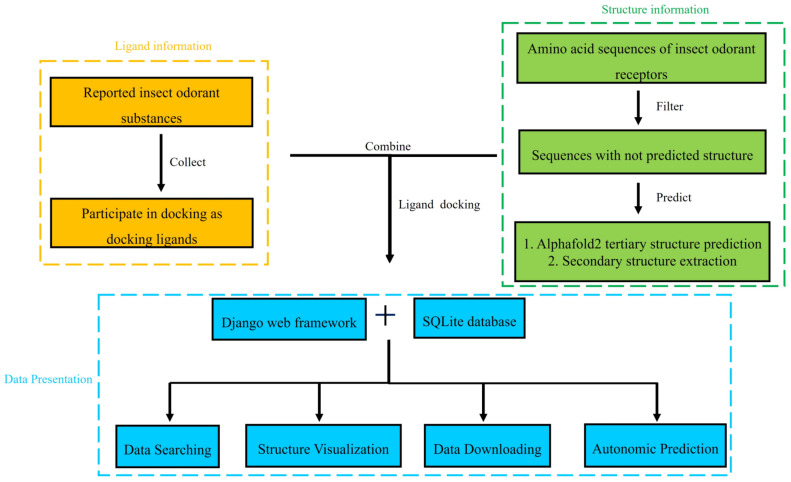
Overview of iORandLigandDB. Ligand information was collected from literature. Structure information was predicted by our platform. Two kinds of data are used as input for protein–ligand docking. These data are displayed in iORandLigandDB built with Django 3.0.4 web framework and all data are stored in an SQLite 3.36.0 database. Users can search, visualize, and download these data, and submit sequences for three-dimensional structure prediction and docking with ligands.

The database was built with Django 3.2.9 web framework, and all data were stored in an SQLite 3.36.0 (https://www.sqlite.org/, accessed on 10 September 2022) database on a Centos 7.6.1810 web server. The web interface is serviced by uWSGI v2.0.20 (https://nginx.org/, accessed on 15 September 2022) and Daphne v3.0.2 (https://github.com/django/daphne/, accessed on 15 September 2022) after Nginx v1.20.1 (https://nginx.org/, accessed on 3 October 2022) proxy. The web template uses the Bootstrap framework (https://getbootstrap.com/, accessed on 5 October 2022), jQuery (https://jquery.com/), and JavaScript (https://www.javascript.com/, accessed on 8 October 2022) to create a user-friendly front-end interface. In addition, Celery-Progress v0.1.2 (https://github.com/czue/celery-progress, accessed on 10 October 2022) and Chart.js (https://www.chartjs.org/, accessed on 10 October 2022) were used to build the progress bar of back-end prediction task and database statistics to enhance the interaction between front and back end (Figure 1).

## 3. Results

### 3.1. Insect Odorant Receptor Sequences

In total, 4426 OR sequences were obtained and classified into 145 species. For each OR sequence, a total of 19 types of information are displayed, including Nucleotide ID, Protein ID, Name, Organism, Description (such as gene name and completeness), Odors, Location, Gene Source, GeneBank Link, Papers, PDBlink, Secondary Structure, Protein, Molecular Weight, Instability Index (the instability index calculates an estimate of the stability of the protein in a test tube [49]), Isoelectric Point (the isoelectric point (pI) is defined as the pH at which the protein/peptide has a net of charge zero [50]), Nucleotide, Structural Information (secondary and three-dimensional), and docking information of 46 ligands and the evaluation parameters of these kinds of structural information.

For each ligand used for docking, 12 types of information are provided, including Database Abbr, Name, PubChem CID, Molecular Weight, Type, Formula, InChI (InChI is a structure-based identifier, strictly unique, and non-proprietary, open source, and freely accessible), InChI Key (InChIKey is a hashed version of InChI which allows for a compact representation and for searching in standard search engines), Canonical SMILES (a unique SMILES string of a compound, generated by a “canonicalization” algorithm), Synonyms, and secondary structure and three-dimensional structure information.

### 3.2. Three-Dimensional Structure of Odorant Receptor Sequences

To make the prediction practically useful, there must be a well-calibrated and sequence-resolved confidence measure. AlphaFold can produce a per-residue confidence metric called the predicted local distance difference test (pLDDT) on a scale from 0 to 100. pLDDT can be used to estimate how well the prediction agrees with an experimental structure based on the local distance difference test Cα (lDDT-Cα) [51], and a higher value indicates better performance [15,52].

**Figure 2:**
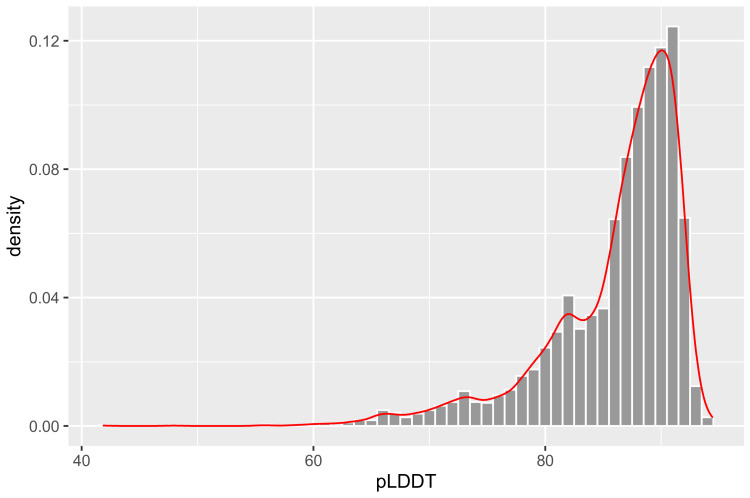
pLDDT of ORs in iORandLigandDB. Most of the pLDDT values are concentrated between 80 and 100.

In total, 4426 protein three-dimensional structures were predicted by Alphafold2. The output results (Figure 2) showed that there were 1165 global pLDDT values above 90, 2665 global pLDDT values within 80–90, 478 global pLDDT values within 70–80, and 118 global pLDDT values below 70, which account for 26.32%, 60.21%, 10.80%, and 2.67% of the ORs, respectively. The pLDDT values of most predicted three-dimensional structures were above 80 and our predicted structures are also similar to many experimental resolved structures and predicted structures [17,53], which showed good confidence and could be used for further research (see Table S3 for details).

### 3.3. Virtual Screening of Ligands

To further analyze the three-dimensional structure and function of ORs, the binding pocket of the proteins was predicted using fpocket v4.0. The pocket center, side length, and volume of the docking box from the output pocket co-ordinates were calculated, except for five sequences for which the binding pocket could not be predicted. The size of the docking box of 4421 sequences ranged from 184.11 to 7615.10 ${Å}^{3}$, and was mainly concentrated in 1363.35–3867.20 ${Å}^{3}$ (see Table S4 for details.)

**Figure 3:**
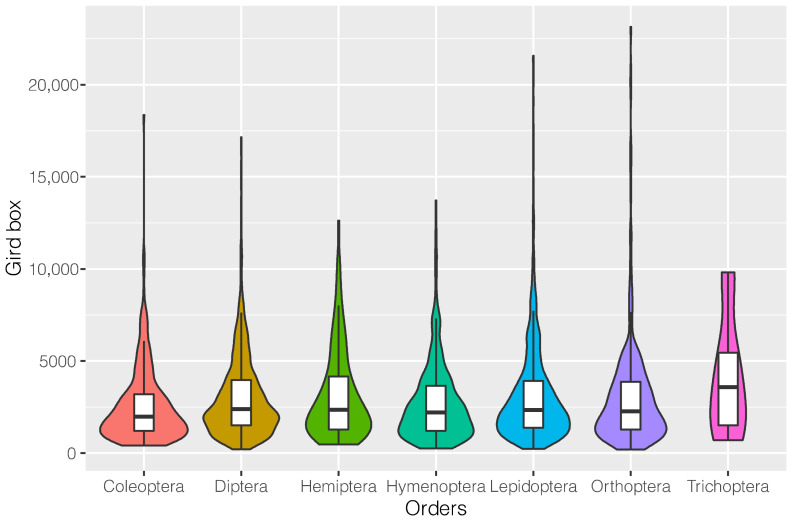
Summary of docking box sizes.

We then divided all the sequences into seven orders according to species, including Coleoptera, Diptera, Hemiptera, Hymenoptera, Lepidoptera, Orthoptera, and Trichoptera (Figure 3). Statistics was performed on the size of docking boxes whose sequences have been reported in the literature. The statistical results showed that the docking box of each order has a range of sizes, which are 414.27–6427.74 ${Å}^{3}$ (mainly between 1227.73 and 3366.13 ${Å}^{3}$) for Coleoptera, 200.88–7615.10 ${Å}^{3}$ (mainly between 1502.20 and3950.45 ${Å}^{3}$) for Diptera, 470.86–6831.30 ${Å}^{3}$ (mainly between 1128.84 and 3737.00 ${Å}^{3}$) for Hemiptera, 334.59–7723.05 ${Å}^{3}$ (mainly between 1386.98 and 3924.36 ${Å}^{3}$) for Lepidoptera, 189.11–7671.45 ${Å}^{3}$ (mainly between 1270.55 and 3891.34 ${Å}^{3}$) for Orthoptera, and 696.40–5825.97 ${Å}^{3}$ (mainly between 1234.29 and 5825.97 ${Å}^{3}$) for Trichoptera. In addition, although there were great variations in the size range of the binding cavity of a specific order, the binding cavity of most odorant-binding proteins did not differ significantly between different orders. Moreover, since each species has a certain olfactory preference, the outliers are likely the specific olfactory preference of some species.

Subsequently, 46 ligands were used as a library to perform the virtual screening of the 4421 predicted proteins. The binding energy for docking was recorded. Theoretically, the docking process with a binding energy lower than zero can occur spontaneously. Therefore, we counted the docking processes with a binding energy below zero, and then selected the docking process with the highest absolute value (the examples of the generated ligand–protein structures can be found in Figure S1).

We found that beta-Caryophyllene had the lowest average binding energy (−9.99 kcal/mol) among the 46 ligands, and Methyl isothiocyanate had the highest average binding energy (−2.53 kcal/mol) (see Table S5 for details). Beta-Caryophyllene is associated with odorant recognition, which has been often reported in important *Lepidoptera* pests such as *Spodoptera littoralis*, *Spodoptera exigua*, *Helicoverpa armigera*, and *Eriocrania semipurpurella* [54,55,56,57], important *Diptera* pests such as *Bactrocera dorsalis* and *Aedes aegypti* [58,59], *Hymenoptera* insects such as *Anastatus japonicas* [60], *Coleoptera* insects such as *Calosoma maximoviczi* and *Anthonomus rubi* [61,62], and *Hemiptera* pests such as *Adelphocoris lineolatus*, *Apolygus lucorum*, and *Diaphorina citri* [63,64,65]. Methyl isothiocyanate is an important novel soil fumigant pesticide [66,67], which showed a low binding degree as expected. In addition, the ligand 2-heptanone with the most broadly known detection range in *Drosophila* also appeared in the database, which is consistent with the report [68].

**Figure 4:**
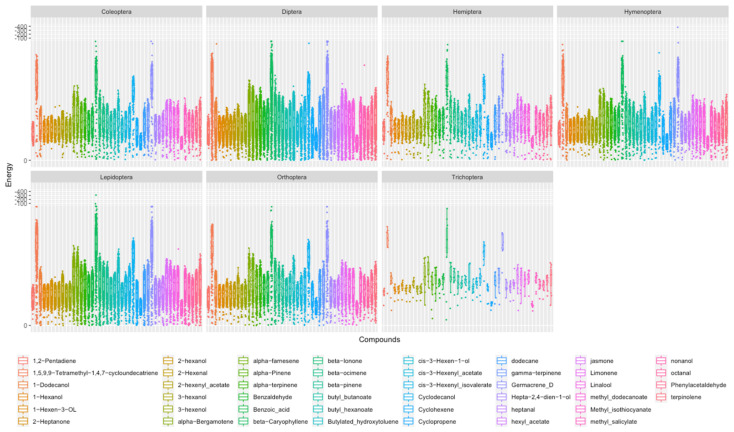
Docking affinity of ORs with ligands in Coleoptera, Diptera, Hemiptera, Hymenoptera, Lepidoptera, Orthoptera, and Trichoptera. The serial number is marked in reference to Table 1 (the docking binding energy diagram for each compound can be found in Figure S2).

From a taxonomic point of view, the seven orders surprisingly exhibited similar energy trends of ligand docking (Figure 4), all of which had four important ligands (average binding energy lower than −6 kcal/mol), including 1,5,9,9-Tetramethyl-1,4,7-cycloundecatriene, beta-Caryophyllene, Cyclodecanol, and Germacrene D. In addition, 1,5,9,9-Tetramethyl-1,4,7-cycloundecatriene has been reported as a component of plant essential oils and is moderately toxic to storage insects [69]. Germacrene D has been reported to induce *Coleoptera* and *Hymenoptera* and is highly specific for the olfactory neurons of *Noctuidae* such as *Helicoverpa armigera*, and is also associated with *Lepidoptera* oviposition and other behaviors [70,71,72].

From the taxonomic perspective of species (Table S2), at the threshold level of <−300 kcal/mol, the binding energy of Germacrene D to iORL002865 (*Campoletis chlorideae*) is −376.00 kcal/mol, and that of beta-Caryophyllene to iORL002782 (*Ostrinia nubilalis*) is −309.20 kcal/mol. At the threshold level of <−50 kcal/mol, the binding energy of beta-Caryophyllene to iORL003762 (*Helicoverpa armigera*) is −90.16 kcal/mol. At the threshold level of <−20 kcal/mol, the binding energy of beta-Caryophyllene to iORL003789 (*Peridroma Saucia*), iORL002781 (*Bactrocera Minax*), iORL002475 (*Drosophila Simulans*), iORL001187 (*Drosophila Simulans*), and iORL002006 (*Campoletis chlorideae*) is −28.73, −23.08, −20.6, −20.5, and −20.25 kcal/mol, and that of Germacrene D to iORL003789 (*Peridroma Saucia*) and iORL002475 (*Drosophila Simulans*) is −28.73 and −23.88 kcal/mol, respectively. These ligands may be important for the corresponding proteins.

In general, the screening results suggested that beta-Caryophyllene, Germacrene D, 1,5,9,9-Tetramethyl-1,4, 7-cycloundecatriene, and Cyclodecanol are related to the odorant recognition of insects. Other odorants with high binding energy have been reported in the corresponding order, and they are also likely important ligands for the corresponding species.

### 3.4. Binding Regions and Transmembrane Domains

In total, we aligned the transmembrane regions of 4421 sequences to the predicted pockets. Our statistics show that ligand-binding regions with a total of 2334 sequences are considered to be located on the transmembrane domain; 1518 are considered to be located within the membrane; and 569 are considered to be located outside the membrane (see Table S6 for details; transmembrane domain amino acid sites are also recorded).

### 3.5. Verification of Docking Posture

The RMSD cutoff of 2 Å is often used as a criterion of the correct bound structure prediction [46]. Verification results showed that 56.00% of the data had RMSD values less than 1 Å, 86.00% less than 2 Å, and 98% less than 3.7 Å. An outlier may be due to a docking error (see Annex Tables S7 and S8 for details). This shows that our method has a certain reliability [73,74]. Therefore, we believe that our predictions can show the correct docking posture.

### 3.6. Web Interface and Usage

**Figure 5:**
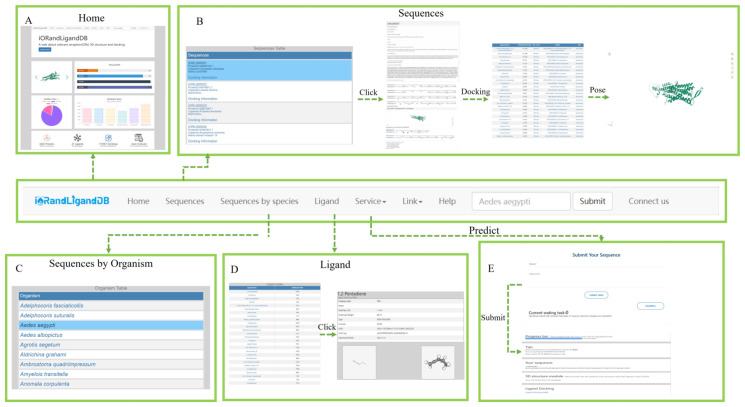
Usage instructions of iORandLigandDB: (**A**) home page; (**B**) protein basic information, protein structure, and docking information page; (**C**) species classification page; (**D**) ligand information page; and (**E**) autonomic prediction page.

iORandLigandDB provides the user-friendly and easy search, browsing, and download of protein-structure and ligand-docking data related to insect ORs. It provides the users with a web interface to predict the protein structure and perform the virtual screening of ligands freely. The top navigation bar includes eight modules, namely, ‘Home’, ‘Sequences’, ‘Sequences by Organism’, ‘Ligand’, ‘Service’, ‘Link’, ‘Help’, and ‘Connect us’ (Figure 5).

The ‘Home’ module provides a quick search of sequences, an introduction to iORandLigandDB, images of protein structures, and statistical tables of database information. The ‘Sequences’ module displays the brief information on sequences. A clicking of the sequence name will display detailed information including basic information, predicted structure information (secondary and three-dimensional), and docking data. The ‘Sequences by Organism’ module divides the sequences based on species, which can be browsed and downloaded according to species. The ‘Ligand’ module introduces the ligand information of the database. The ‘Service’ module has two functions: ‘Protein Prediction’ allows the user to submit the odorant sequence autonomously. After a successful submission, the three-dimensional structure will be predicted automatically, and ligand docking will be completed. The resulting file will be returned to the sub-interface, and the task progress, three-dimensional structure, and docking data will be visualized. The ‘Submit Your OR sequence’ module allows users to submit their OR sequences to the database. The ‘Link’ module provides links to other databases. The ‘Help’ module describes the usage, and the ‘Connect us’ module describes the contact information.

The ‘Home’ page provides a summary of database statistics presented in the form of diagrams. The page can help users to have an intuitive understanding on the database. The ‘Sequences’ page provides a list of database sequences. The main page lists the brief information of the sequences. The secondary page provides the basic information. The tertiary page provides a list of binding affinities of the ligand, and every docking posture can be viewed. Users can browse and download this information freely. The ‘Sequences by species’ page provides a list of sequences classified according to species. Users can download and browse the information by species. The ‘Ligand’ page provides a list of ligands used in database. The main page lists the ligand name and its abbreviation in the database. The secondary page provides the basic information and visualized two- and three-dimensional structure.

## 4. Discussion

The development of computer technology and artificial intelligence algorithms facilitates the more accurate inference of the spatial structure of a protein based on its primary structure. Clarifying the functions of key ORs in insect olfaction can help to infer the relevant substances to which insects are sensitive, which can then be used to develop attractants or repellents. The iORandligandDB website provides an easy-to-use web computing platform. Researchers can search for insect-specific odorants already present in the server or submit their own insect OR sequences to the service platform for the three-dimensional structure prediction, molecular docking, and preliminary acquisition of insect-specific odorants. The website is expected to save researchers a lot of work when studying insect ORs to explore insect-specific odorants.

## Figures and Tables

**Table 1:** The database number corresponds to the ligand name and abbreviation.

Id	Name	Abbreviation
1	1,2-Pentadiene	PEN
2	2-Hexenal	HX2
3	beta-Caryophyllene	CAY
4	Linalool	LIN
5	1,5,9,9-Tetramethyl-1,4,7-cycloundecatriene	TEC
6	alpha-Bergamotene	BER
7	beta-Ionone	ION
8	Cyclohexene	CYC
9	Methyl isothiocyanate	MEI
10	1-Dodecanol	DOD
11	alpha-Farnesene	FAR
12	Butylated hydroxytoluene	BUH
13	Cyclopropene	CYL
14	Phenylacetaldehyde	PHE
15	1-Hexanol	HE1
16	alpha-Pinene	PIN
17	cis-3-Hexen-1-ol	CHO
18	Germacrene D	GED
19	1-Hexen-3-OL	HEO
20	Benzaldehyde	BEN
21	cis-3-Hexenyl acetate	CHA
22	Hepta-2,4-dien-1-ol	HDO
23	2-Heptanone	HEP
24	Benzoic acid	BEA
25	cis-3-Hexenyl isovalerate	CHI
26	Limonene	LIM
27	Cyclodecanol	CYO
28	alpha-terpinene	TER
29	jasmone	JAS
30	methyl_dodecanoate	MDO
31	2-hexenyl_acetate	HEX
32	dodecane	DOE
33	methyl_salicylate	MES
34	2-hexanol	HEA
35	hexyl_acetate	HET
36	3-hexenol	HX3
37	terpinolene	TEP
38	3-hexanol	HEN
39	octanal	OCT
40	nonanol	NOA
41	heptanal	HEL
42	butyl_butanoate	BUB
43	beta-pinene	BPI
44	butyl_hexanoate	BUE
45	gamma-terpinene	GAM
46	beta-ocimene	OCI

## Data Availability

iORandLigandDB is freely available at https://www.iorandliganddb.com/ (access on 21 May 2023).

## Supplementary Materials

The following supporting information can be downloaded at: https://www.mdpi.com/article/10.3390/insects14060560/s1, Figure S1: Examples of the generated ligand-protein structures; Figure S2: Docking affinity of ORs with ligands in *Coleoptera*, *Diptera*, *Hemiptera*, *Hymenoptera*, *Lepidoptera*, *Orthoptera*, and *Trichoptera*; Table S1: Database static; Table S2: Organism static; Table S3: pLDDT static; Table S4: Girdbox static; Table S5: Affinity static; Table S6: TM static; Table S7: PDB sequences source table; Table S8: RMSD statistics.
