# Supplementary figures and images for: iORandLigandDB: A Website for Three-Dimensional Structure Prediction of Insect Odorant Receptors and Docking with Odorants
Source: Insects. 2023 Jun 15;14(6):560. doi: 10.3390/insects14060560 (PMC10299237; doi:10.3390/insects14060560)

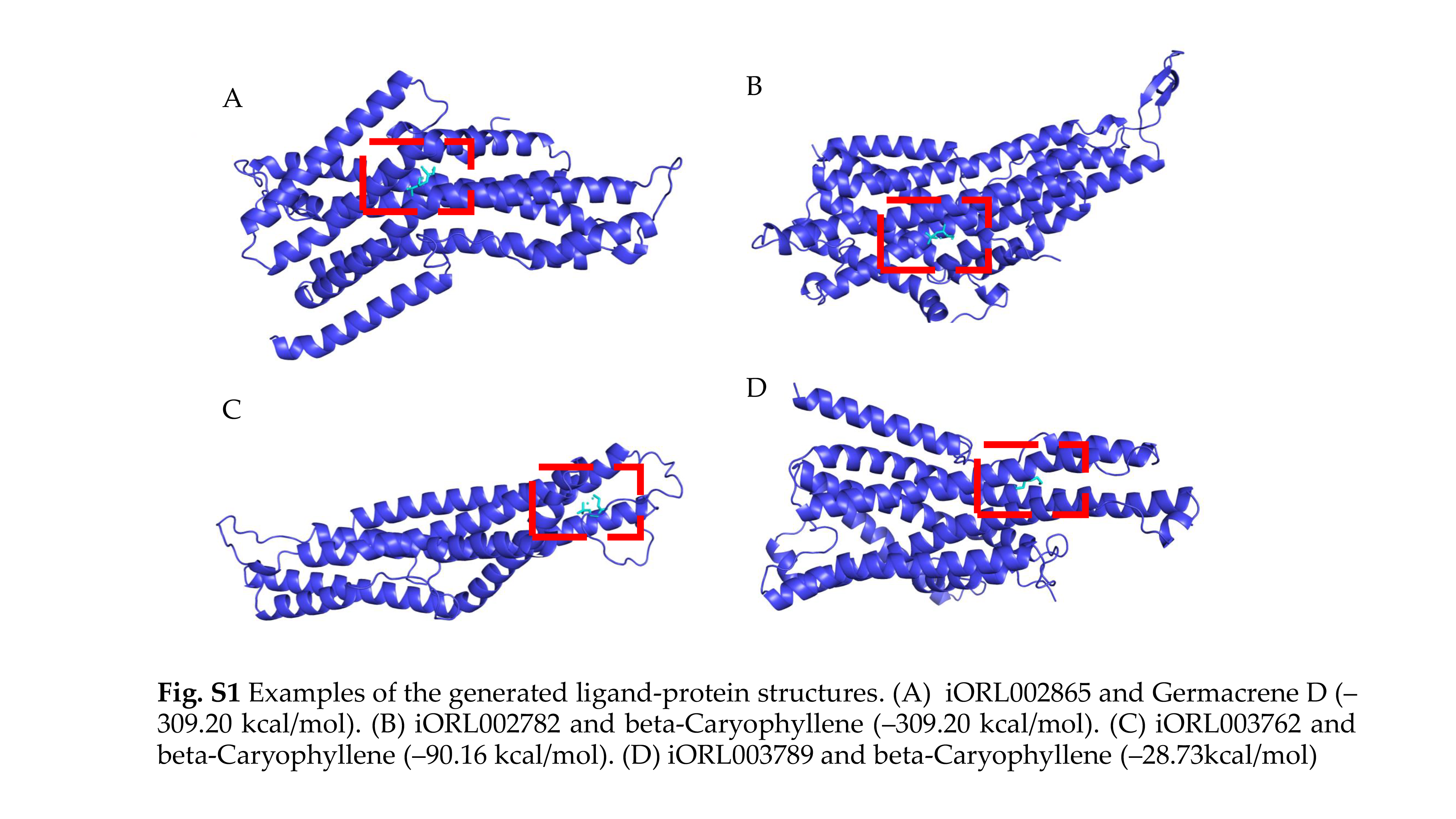

Supplement: Supplementary file 1 [file insects-14-00560-s001.zip › insects-2353674-supplementary-conversion/insects-2353674-supplementary/Figure S1 Examples of the generated ligand-protein structures.tif]
